# Proteomic Perspective of Cadmium Tolerance in *Providencia rettgeri* Strain KDM3 and Its *In-situ* Bioremediation Potential in Rice Ecosystem

**DOI:** 10.3389/fmicb.2022.852697

**Published:** 2022-04-26

**Authors:** Darshana A. Salaskar, Mahesh K. Padwal, Alka Gupta, Bhakti Basu, Sharad P. Kale

**Affiliations:** ^1^Nuclear Agriculture and Biotechnology Division, Bhabha Atomic Research Centre, Mumbai, India; ^2^Molecular Biology Division, Bhabha Atomic Research Centre, Mumbai, India; ^3^Applied Genomics Section, Bhabha Atomic Research Centre, Mumbai, India

**Keywords:** cadmium, *Providencia rettgeri*, *Oryza sativa*, bioremediation, proteomics

## Abstract

In this study, a multi-metal-tolerant natural bacterial isolate *Providencia rettgeri* strain KDM3 from an industrial effluent in Mumbai, India, showed high cadmium (Cd) tolerance. *Providencia rettgeri* grew in the presence of more than 100 ppm (880 μM) Cd (LD_50_ = 100 ppm) and accumulated Cd intracellularly. Following Cd exposure, a comparative proteome analysis revealed molecular mechanisms underlying Cd tolerance. Among a total of 69 differentially expressed proteins (DEPs) in Cd-exposed cells, *de novo* induction of *ahpCF* operon proteins and L-cysteine/L-cystine shuttle protein FliY was observed, while Dps and superoxide dismutase proteins were overexpressed, indicating upregulation of a robust oxidative stress defense. ENTRA1, a membrane transporter showing homology to heavy metal transporter, was also induced *de novo*. In addition, the protein disaggregation chaperone ClpB, trigger factor, and protease HslU were also overexpressed. Notably, 46 proteins from the major functional category of energy metabolism were found to be downregulated. Furthermore, the addition of *P. rettgeri* to Cd-spiked soil resulted in a significant reduction in the Cd content [roots (11%), shoot (50%), and grains (46%)] of the rice plants. Cd bioaccumulation of *P. rettgeri* improved plant growth and grain yield. We conclude that *P. rettgeri*, a highly Cd-tolerant bacterium, is an ideal candidate for *in-situ* bioremediation of Cd-contaminated agricultural soils.

## Introduction

Heavy metals are metallic elements with relatively high densities and are toxic at low concentrations. Historically, anthropogenic activities such as ore mining and industrialization are directly associated with environmental pollution with heavy metals worldwide (Ozaki et al., 2019; Hubeny et al., 2021). Since the heavy metals are naturally present in the earth's crust, they cannot be destroyed or degraded, but can only be transformed into a less toxic form (González Henao and Ghneim-Herrera, 2021). They enter the human body *via* polluted air, contaminated food, or drinking water. While a few are essential in trace quantities as cofactors for different enzymes, the rest are non-essential. Nonessential heavy metals, including cadmium (Cd), are especially concerning as they pose a serious threat to human life (Rafati Rahimzadeh et al., 2017).

Cadmium is the 7th most toxic element (Fay and Mumtaz, 1996); however, it has a prolonged biological half-life of about 10–30 years (Berglund et al., 2015). At the molecular level, Cd induces reactive oxygen species (ROS) that further cause DNA damage and mutations, disrupt RedOx potential, inactivate the antioxidant enzymes, culminating in disruptions of a wide range of cellular functions such as respiration, oxidative phosphorylation, cell proliferation, etc. (Rafati Rahimzadeh et al., 2017). Some of the reported deleterious manifestations of Cd poisoning include lung damage, renal dysfunction, and bone toxicity (Kaji, 2012). A plant-based diet is the predominant route of entry of Cd into the food chain. Rice is an economically important crop in Asia and the staple food for over half of the world's population (Wang et al., 2019; Zhao and Wang, 2020). However, it is a major source of both arsenic (As) and Cd since rice plants have a higher ability to accumulate these heavy metals compared to other cereal grains (Sui et al., 2018). Since Cd(II) ionic form of Cd is highly soluble in soil, it is the most predominant toxic metal transferred from soil to food, especially in the rice grains (Zhao and Wang, 2020). Cd-contaminated rice fields reportedly caused itai-itai disease in Japan in the last century (Kaji, 2012).

In recent years, microbial remediation strategies have attracted increasing attention since they are efficient, cost-effective, sustainable, and environmentally friendly (Volesky and Holan, 1995; González Henao and Ghneim-Herrera, 2021). Microbial remediation of heavy metals consists of biosorption, bioaccumulation, biomineralization, and biotransformation (Ayangbenro and Babalola, 2017). Microbes that possess molecular mechanisms to tolerate the toxic effects of heavy metals are especially useful for heavy metal remediation. Among the 5 main resistance mechanisms reported (González Henao and Ghneim-Herrera, 2021), intracellular sequestration remains most relevant to the Cd remediation of paddy to limit the availability of Cd to the rice plants. A variety of biomass, including microbes, yeast, fungi, and plants, have been successfully used for the remediation of heavy metals from contaminated habitats (Kumar et al., 2019; Rahman, 2020; Wei et al., 2021). Conversely, such contaminated habitats are a useful resource for native flora with heavy metal resistance (Rahman, 2020; Wei et al., 2021). We have earlier reported the identification and characterization of *Providencia rettgeri* strain KDM3 isolated from metal-contaminated soil from industrial premises in Mumbai, India (Salaskar, 2015). The organism showed tolerance to high concentrations of arsenic and resistance to multiple antibiotics (Salaskar, 2015). Antibiotic and metal resistance determinants are often co-selected (Baker-Austin et al., 2006), and different *Providencia* species show tolerance to multiple heavy metals (Sharma et al., 2017; Adekanmbi et al., 2019; Shukla et al., 2021). However, the scope of heavy metal resistance in the natural isolate *P. rettgeri* KDM3 remains unexplored.

In this study, we report the resistance of the bacterial strain *P. rettgeri* KDM3 to high concentrations of Cd. Intracellular accumulation of Cd was confirmed by electron microscopy and elemental analysis. Furthermore, we investigated the determinants of Cd tolerance in *P. rettgeri* KDM3 using a proteomic approach. The first 2D proteome map of *P. rettgeri* KDM3 was generated with 147 proteins identified by mass spectrometry. Comparative proteomics revealed that the strain upregulated oxidative stress defense, membrane transport, and protein homeostasis pathways to survive Cd exposure at the expense of downregulation of various metabolic pathways. Bioaugmentation studies showed significant improvements in the growth parameters of the rice plants exposed to 100 ppm Cd and a remarkable reduction in the Cd concentration in the edible parts of the rice plant. To the best of our knowledge, this is the first study where the beneficial effect of *P. rettgeri* KDM3 is systematically assessed and till the maturation stage (135 days) of the rice plant. The organism presents great potential for *in-situ* Cd bioremediation in paddy fields.

## Materials and Methods

### Bacterial Strain, Growth Conditions, and Cd Exposure

Isolation, characterization, and identification of bacterial isolate *P. rettgeri* strain KDM3 (Genbank Accession Number: KC247668.1, GI: 432140652) have been described earlier by Salaskar (2015). For various assays, *P. rettgeri* strain KDM3 cells were resuspended in fresh lysogeny broth (LB) medium at a starting cell density of OD_530nm_ = 0.05, and the cells were allowed to grow under standard growth conditions. When the cell density reached OD_530nm_ = 0.5, various Cd stress assays, such as growth experiments, microscopic investigations, and proteomic and rice ecosystem studies, were performed using CdCl_2_.2½ H_2_O salt. *P. rettgeri* cells in the exponential phase (OD_530_ = 1.0–1.2) were resuspended in the LB broth containing 0–250 μg Cd ml^−1^ (0–250 ppm) and incubated at 30°C in an orbital shaker (140 rpm). The growth of *P. rettgeri* was monitored at various time intervals till 8 h by measuring the optical density of the culture at 530 nm using UV/Vis spectrophotometer (Jasco-V-530). Plasmid isolation and plasmid curing experiments were performed as per the protocol detailed earlier (Salaskar, 2015).

### Cd Concentration Measurements

The concentration of Cd accumulated by *P. rettgeri* was measured after 20 h of exposure to 0–250 ppm Cd. The bacterial cells were spun down (10,000 g, 10 min) to remove residual Cd and resuspended in 10 ml of distilled water, vortexed, and centrifuged at 10,000 g, 10 min. After centrifugation, the bacterial cell pellet was digested in a microwave digestion system (Milestone Ethos up, Italy) using 6 ml concentrated HNO_3_. The cooled digested solution was further concentrated to 2 ml and diluted to 10 ml with water, and the Cd concentration was measured using a Flame Atomic Absorption Spectrometer (GBC 932 plus, Australia). The detection limit of Cd was 0.2–1.8 ppm, the sensitivity was 0.009 ppm, and the wavelength was set at 228.8 nm with D2 correction. The assay was performed in triplicates.

### Microscopic Investigations and Elemental Mapping

*Providencia rettgeri* cells were exposed to 100 ppm Cd for 20 h. Following Cd exposure, the cells were harvested, washed, and observed under a light microscope (Carl Zeiss Axiostar plus) with a 100 × oil immersion objective under hydrous conditions. *Providencia rettgeri* cells were processed similarly, except that exposure to Cd served as control. For scanning electron microscopy (SEM), the cells were washed in normal saline and fixed in 2.5% glutaraldehyde for 2 h. The cells were then dehydrated in a graded ethanol series (30–100%), spotted on aluminum studs, and dried at 37°C for 1 h. The dried samples were gold coated using the thermal evaporation technique and analyzed by SEM using a Tescan VEGA 40 Microscope. For transmission electron microscopy (TEM), cells were washed in normal saline and were fixed with 2.5% glutaraldehyde, a 0.5% paraformaldehyde mixture, dehydrated with increasing concentrations of ethanol, and embedded in epoxy resin. Then sections (50 nm) were cut and visualized under Carl Zeiss Libra 120 keV TEM without staining. Energy-filtered transmission electron microscopy (EF-TEM) was used for elemental mapping on the electron-dense area as described earlier (Saunders and Shaw, 2014; Anaganti et al., 2015), except that the slit width of the Cd-specific energy filter was set at 390 and 503 ev.

### 2D Gel Electrophoresis and Image Analysis

Cellular proteins were resolved by 2D gel electrophoresis as described earlier (Basu and Apte, 2012). In brief, the cellular protein samples were prepared from control or Cd-treated cells following 1 h of exposure to 100 ppm (880 μM) Cd. The cup loading method was used to resolve cellular proteins (500 μg) by isoelectric focusing (17 cm IPG strip, pI 4–7, Bio-Rad, India), followed by 12% SDS-PAGE. The gels were stained with Coomassie Brilliant Blue G250 stain and digitized using a Dyversity-6 gel imager (Syngene, UK). Three biological replicate sets were generated by repeating the experiment three times. PDQuest 2D analysis software (version 8.1.0, Bio-Rad) was used for the first level match set generated from three biological 2D gel replicates. The minimum correlation coefficient value between the replicates of group sets was 0.75. Protein spots were detected and matched between the replicates in automatic detection mode, and spuriously detected spots were removed manually. Spot densities were normalized with the local regression method. An independent Student's *t*-test was applied for statistical analysis and the protein spots with *p*-values ≤ 0.05 were considered as significantly differentially expressed between control and Cd-treated cells. The protein spots of interest were manually excised from the gel and further identified by mass spectrometry.

### Mass Spectrometry and Protein Identification

The protein gel plugs were destained, reduced with Dithiothreitol (DTT), alkylated with iodoacetamide, in-gel digested with trypsin, and the oligopeptides were eluted as described earlier (Anaganti et al., 2015). Similarly, co-crystallization of the eluted oligopeptides and mass spectrometric (UltraFlex III MALDI-TOF/TOF mass spectrometer, Bruker Daltonics, Germany) analysis were performed as described earlier (Anaganti et al., 2015). Mascot searches were conducted using the NCBI nonredundant database (released in January 2016 or later, with a minimum of 79,354,501 entries actually searched) with the following settings: the number of missed cleavages permitted was 1; fixed modifications such as carbamidomethyl on cysteine, variable modification of oxidation on methionine residue; peptide tolerance as 100 ppm; enzyme used as trypsin; and a peptide charge setting as +1. *Providencia rettgeri* protein with significant Molecular Weight Search (MOWSE) score considered as successful identification.

### Bioinformatics Analyses

The hierarchical cluster was created using the pheatmap toolbox in RStudio (Kolde, 2019). Briefly, raw protein intensity values were imported into the RStudio, row scaled, and plotted using default parameters. Gene Ontology (GO) enrichment analysis was carried out using the Bingo tool in Cytoscape (Maere et al., 2005). For this purpose, a basic ontology file in Open Biomedical Ontologies (OBO) format was downloaded from the GO website (http://geneontology.org/). *Providencia rettgeri*-specific annotations were retrieved from the Quickgo website (https://www.ebi.ac.uk/QuickGO/) using taxon ID filter (587). Protein identities were mapped to the corresponding UniProt ID for *P. rettgeri* using the UniProt ID-conversion tool. GOs were FDR corrected, and the selected ontology was plotted in RStudio using “ggplot2” for representation (Wickham, 2016).

### *In-situ* Bioremediation Studies Using Model Rice Ecosystem

A greenhouse glass pot culture experiment was conducted using an agriculture acidic soil (pH, 5.9) in Dapoli, Maharashtra (Table 1). The soil was passed through a 2-mm sieve. Glass tanks (60 cm in diameter and 30 cm in height) were filled with 15 kg of sieved soil, and 60 plants were planted per pot. The experimental sets were (a) control soil with no Cd (C), (b) 100 ppm Cd in soil (T1), and (c) 100 ppm Cd with 1 × 10^8^ cells of *P. rettgeri* strain g^−1^ soil (T2). Three replicates were maintained for each set. The pots were maintained at 60% water holding capacity for 1-week for bacterial soil colonization. The soil was treated with CdCl_2_.2½ H_2_O (solubility in water 135 g/100 ml at 20°C). Bacterial colonization was checked during plant growth and after crop harvest by plating the representative soil samples followed by the serial dilution technique. *Providencia rettgeri* cells were selected on agar plates containing 50 ppm kanamycin.

**Table 1:** Physico—chemical characteristics of the experimental soil.

**Soil details**	
Location	Dapoli-Maharashtra
Color	Red
Class	Inceptisol
**Soil physico-chemical characteristics**	**Values**
pH (soil-water 1:2.5)	5.9
Electrical conductivity (μSm^−1^)	0.11
Cation exchange capacity [c mol (p^+^) kg^−1^]	19.4
Water holding capacity (%)	55
Organic carbon (%)	1.1
Available *P* (μg g^−1^)	35
Total *N* (%)	0.08
*DTPA extract (μg g^**−1**^ **soil)**	
Cd	1.6
Cu	2.72
Fe	18.58
Mn	116.8
Zn	0.32

* *DTPA, DietheyleneTriamine Penta acetic acid*.

### Seed Treatment and Rice Plant Growth

*Oryza sativa* BARC-KKV-13 was used in this study. Seeds were surface sterilized with 0.1% HgCl_2_ for 1 min, washed 3–4 times with running tap water, followed by one wash with distilled water, and dried on the blotting paper. The seedlings were grown in plastic trays containing control soil for about 2 weeks, and later seedlings with similar appearance and biomass were carefully transplanted into the, respectively, developed glass ecosystem as stated above. The rice crop was grown till maturity (135 days). Fertilizers were applied at regular intervals as per standard agriculture practice, and flooding condition was maintained throughout the growth period by replenishing water every 2 days. After 135 days, the plants were carefully removed from the pots by separating the roots and the shoots. Shoot samples were rinsed several times with water to remove surface contamination, and the root surfaces were washed several times with tap water, followed by distilled water, and further with 0.5 mM ethylenediaminetetraacetic acid (EDTA) to remove adhering Cd on the root surface.

### Determination of Plant Fresh Weight, Root Length, Shoot Length, and Cd Concentrations in Plant Parts

The various physiological parameters of plants were determined by measuring the lengths and fresh weights of plant roots, shoots, or grains. The plant shoot, root, and grain samples were dried in a hot air oven at 70°C till a constant weight was achieved, and dry matter yield was recorded. To determine the Cd content, the dried samples were finely ground and wet digested in a microwave digestion system (Milestone Ethos UP, Italy) in concentrated HNO_3_ (AR grade). The cooled digested solution was further concentrated to 2 ml and diluted to 10 ml with water, and the Cd concentration was measured using a flame atomic absorption spectrometer (GBC 932 plus, Australia), as described in the Section “Cd Concentration Measurements.”

### Statistical Analysis

All the experiments were performed in triplicates to generate three biological replicates, and the results have been reported as means ± standard deviation (SD). The software used for statistical analyses was ANOVA in Excel 2007. Statistical analyses for various parameters in the rice ecosystem experiment included comparisons of individual groups using the *post-hoc Q*-test. The *p*-values were determined using a *post-hoc Q* table. The significant difference was determined through Tukey's mean grouping at α = 0.05, i.e., 95% CI, and α = 0.01, i.e., 99% CI.

## Results and Discussions

### Cd Tolerance and Bioaccumulation by *P. rettgeri* Strain KDM3

**Figure 1:**
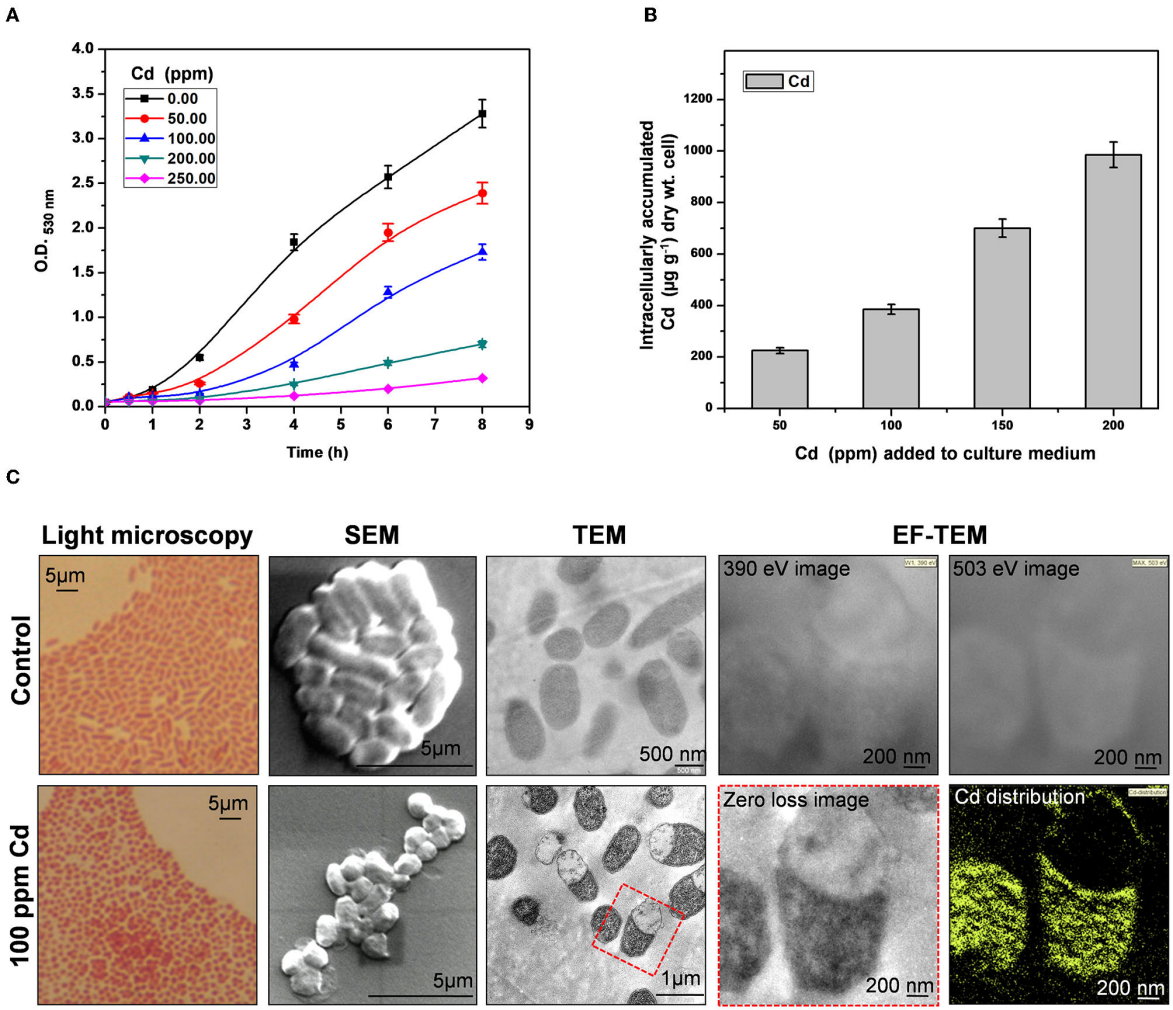
Cadmium (Cd) tolerance and accumulation by *Providencia rettgeri*. **(A)** Growth of *P. rettgeri* in the presence of different concentrations of Cd (0–250 ppm). **(B)** Cd concentration in *P. rettgeri* cells grown in the presence of increasing concentrations of Cd for 20 h. **(C)** Morphology of *P. rettgeri* cells observed under light microscopy, scanning electron microscopy, transmission electron microscopy, and elemental mapping of cadmium using energy-filtered transmission electron microscopy. The *P. rettgeri* cells were either grown in the presence of 100 ppm Cd or not (control). The red dotted box shows the cells on which EFTEM analysis was carried out.

*Providencia* species are tolerant to high concentrations of an array of heavy metals (Thacker et al., 2006; Naik et al., 2013; Salaskar, 2015). We found that arsenic-tolerant *P. rettgeri* strain KDM3 also exhibited remarkable tolerance to high concentrations of Cd. The organism could grow in the increasing concentrations of Cd, while a concentration of 100 ppm Cd (880 μM Cd) resulted in a 50% decrease in growth rate (LD_50_), as compared to control cells (Figure 1A). An increase in Cd content in the media caused a proportionate increase in the Cd uptake by the growing cells (Figure 1B). At 200-ppm Cd exposure, *P. rettgeri* cells accumulated ≈ 1 mg/g Cd/dry weight of cells (Figure 1B). Exposure to Cd (100 ppm) induced morphological changes in *P. rettgeri*. Loosely packed short rods of control cells changed to characteristic chains of coccoidal lenticular shape after being exposed to 100 ppm Cd, as seen under a light microscope as well as SEM (Figure 1C). Transmission electron micrographs showed spread-out electron-dense depositions inside the Cd-exposed cells (Figure 1C), indicating the presence of Cd. The intracellular distribution of Cd was confirmed by the elemental mapping of cells through Energy-Filtered Transmission Electron Microscopy (EFTEM). A large intracellular distribution of Cd was observed in the electron-dense region of Cd-treated cells, which was absent in control cells (Figure 1C). From these data, we concluded that the high Cd tolerance of *P. rettgeri* KDM3 strain is due to intracellular accumulation of the toxic metal.

### Reference Proteome Map of *P. rettgeri* and Functional Classification of the Identified Proteins

**Figure 2:**
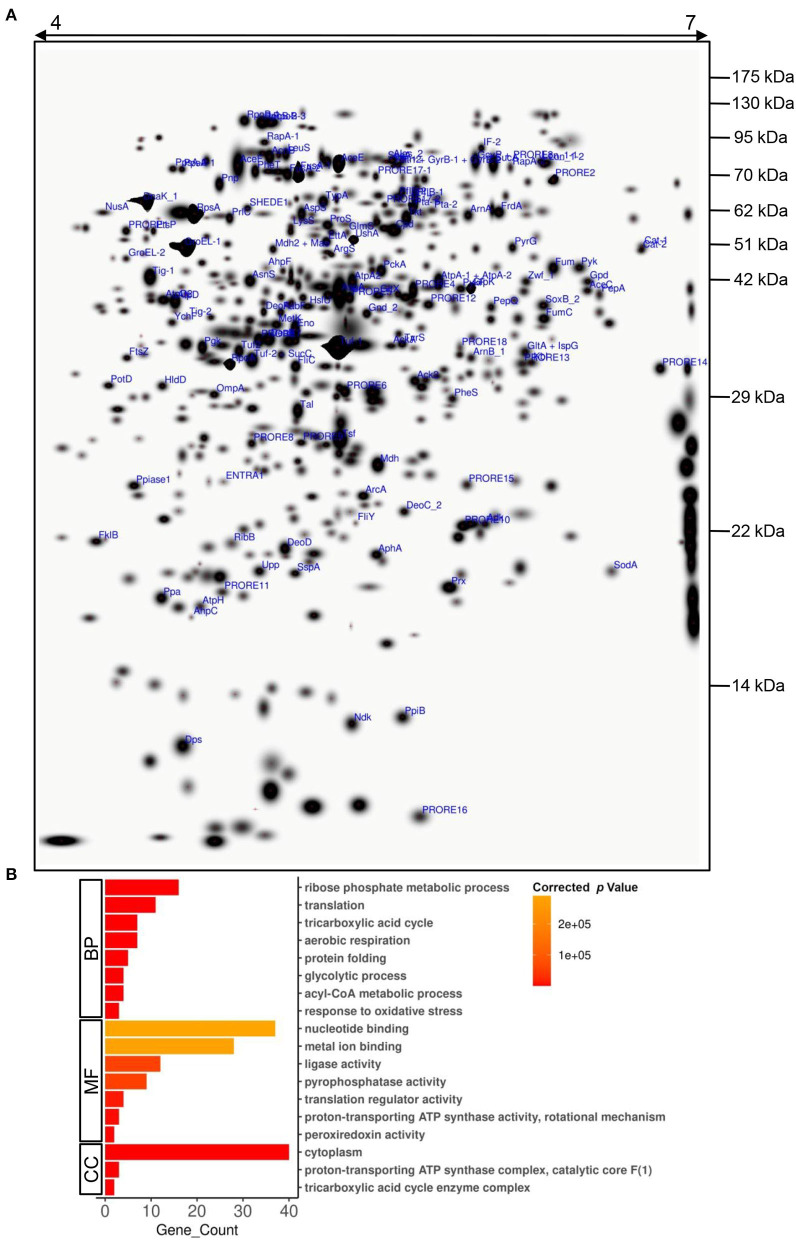
A 2D proteome map of *P. rettgeri*. **(A)** A “master gel” representing a proteome of *P. rettgeri* control cells was created *in silico* from three biological replicate gels. The pI and molecular masses (kDa) of marker proteins are shown at the top and on the right-hand side of the gel, respectively. All the identified proteins are marked. Refer to Supplementary Table S2 for spot ID annotations. **(B)** Gene Ontology (GO) functional classification of identified proteins using BiNGO tool. BP, biological process; MF, molecular function; CC, cellular component.

Even though *Providencia* is known to be able to survive high concentrations of heavy metals, its Cd stress response network has not been explored yet. Plasmid-mediated Cd resistance has been reported for *P. aeruginosa* (Chellaiah, 2018). We earlier found that the arsenic tolerance of *P. rettgeri* strain KDM3 was plasmid-borne (Salaskar, 2015); however, Cd tolerance of the organism was unaffected even after plasmid curing, indicating that the determinants of Cd tolerance were encoded on the chromosome (data not shown). To systematically investigate the response of *P. rettgeri* to Cd exposure through a proteomic approach, we first proceeded to generate the 2D proteome map of *P. rettgeri* since the reference proteome map is not available for any *Providencia* strains yet. The cellular proteins were extracted from *P. rettgeri* cells exposed to 100 ppm Cd (test) for 1 h or not (control) and resolved by 2D gel electrophoresis. On average, PDQuest image analysis software detected about 543 high-quality spots on the 2D gel sets. Among the replicates in each group, high correlations were observed for protein spot matching in the individual group (Supplementary Table S1). Since a 2D proteome map is not yet available for *P. rettgeri*, we picked up a total of 144 protein spots for identification by MALDI mass spectrometry. We identified a total of 147 proteins confidently (Figure 2A, Supplementary Figure S1, Supplementary Table S2, Supplementary MS Data). The identified proteins belonged to 132 unique genes since 15 proteins (isoforms) were identified from more than one spot (Supplementary Table S2).

To decipher the biological processes, molecular functions, and cellular components of the identified proteins, GO enrichment analysis was carried out. Biological processes such as ribose phosphate metabolic process, TCA cycle, glycolytic process, acyl-CoA metabolic process, translation, protein folding, aerobic respiration, and response to oxidative stress were enriched among the identified proteins (Figure 2B, Supplementary Table S3). We also observed enrichment of various molecular functions such as binding of nucleotides or metal ions, translation regulation, ligase activity, pyrophosphatase activity, proton-transporting ATP synthase activity, and peroxiredoxin activity (Figure 2B, Supplementary Table S4). The majority of the identified proteins were cytoplasmic, while a few were part of protein complexes such as ATP synthase complex, TCA cycle enzyme complex, etc. (Figure 2B, Supplementary Table S5).

### Differential Proteome of *P. rettgeri* in Response to Cd Exposure and GO-Based Functional Classification of DEPs

**Figure 3:**
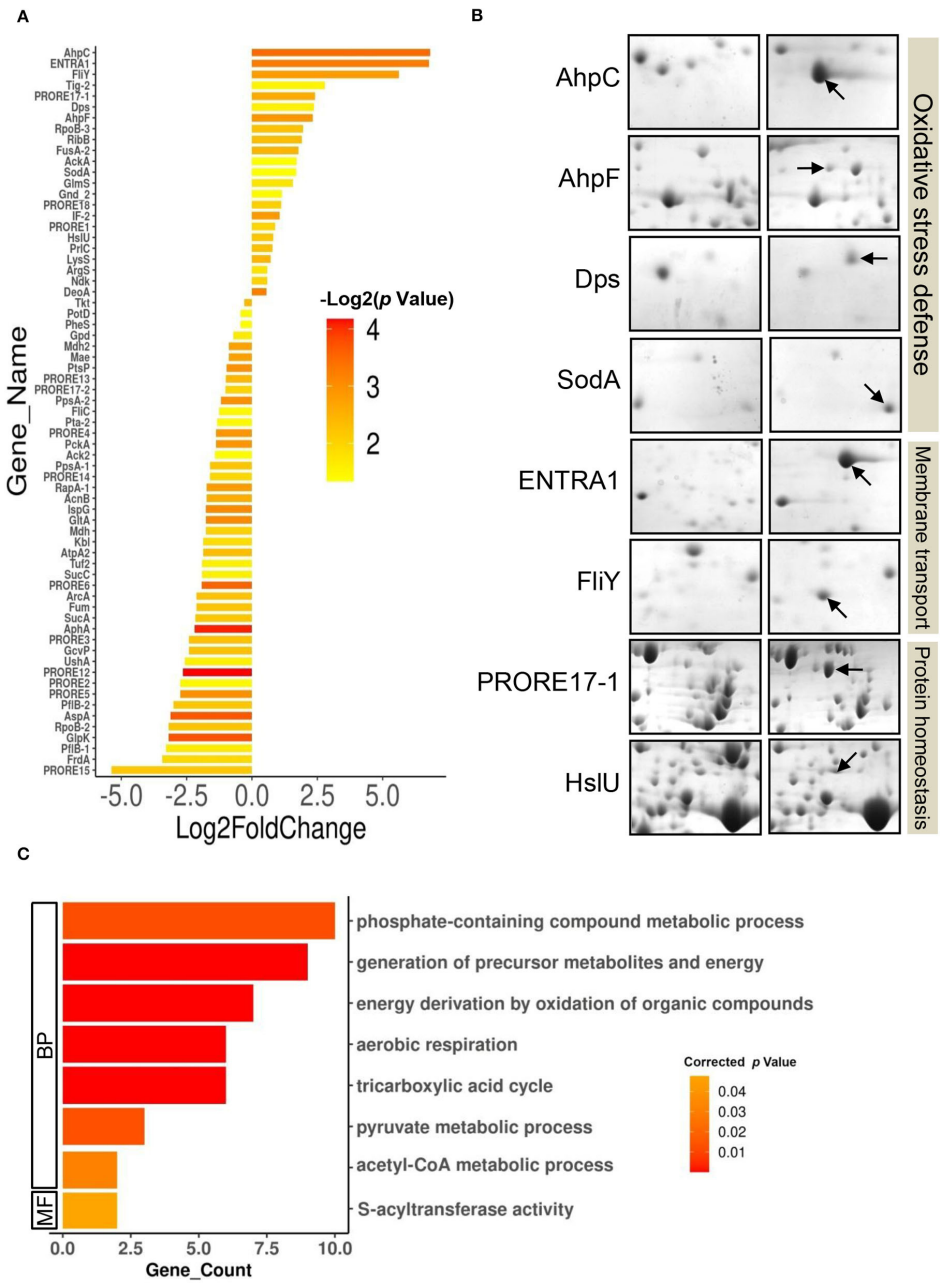
Differentially expressed proteome of *P. rettgeri* exposed to 100 ppm Cd. **(A)** Bar plot representing the Log2 (fold change) on *X*-axis vs. *p*-value for the differentially expressed proteins (DEPs) on *Y*-axis. Log2 (fold change) is calculated by taking the ratio of the mean values of protein intensities from treated and control gels. The *p*-values plotted are calculated from the differential expression analysis using the Student's *t*-test. Bars are colored according to the *p*-values. Protein abbreviations are detailed in Supplementary Table S2. **(B)** *Providencia rettgeri* proteins upregulated following exposure to 100 ppm Cd. Proteins with increased abundance in Cd-treated cells, as compared to control cells, are shown by arrows. **(C)** GO functional classification of downregulated DEPs.

Furthermore, differential expression analysis on the proteome profiles of the control and test cells revealed a set of 69 proteins differentially expressed (*p*-value < 0.05) in response to 100 ppm Cd exposure (Figure 3A, Supplementary Table S6, Supplementary Figure S2). The DEPs belonged to 66 unique genes since three proteins were present in at least two spots. In all, 23 proteins were upregulated while 46 proteins were downregulated following exposure to 100 ppm Cd (Figure 3A, Supplementary Table S6). Among the upregulated proteins, *de novo* expression was observed for the typical bacterial 2-Cys peroxiredoxin AhpC (112.85-fold), while its cognate reductase AhpF showed a 5-fold increase in abundance in the cells exposed to 100 ppm Cd (Figure 3B, Supplementary Table S6). The genes encoding AhpC and AhpF proteins were found to be present in an operonic arrangement on the genome of *P. rettgeri* (Supplementary Figure 3A). Interestingly, the *P. rettgeri* cells retained the Cd tolerance property even when cured of the plasmid, suggesting that the *ahp* operon could be a major determinant of Cd tolerance in this organism. AhpC, belonging to a highly conserved thiol-specific antioxidant (TSA) family, possesses a peroxidase activity against H_2_O_2_, peroxynitrite, and organic hydroperoxides. In a homodimeric AhpC, the peroxidatic cysteine (C-47) is first oxidized to sulfenic acid intermediate and resolved by the cysteine (C-166) in the other subunit. Both the cysteine residues are completely conserved in AhpC encoded by *P. rettgeri* (Supplementary Figure 3B). AhpF acts as a reductase that uses electrons from NADH to reduce intermolecular disulfide bonds in an oxidized AhpC dimer (Zhang et al., 2019; Feng et al., 2020). Other noteworthy upregulated antioxidant enzymes include DNA starvation/stationary phase protection protein Dps (5.2-fold induction) and Mn-Superoxide dismutase (3.2-fold induction) (Figure 3B, Supplementary Table S6).

We also observed the *de novo* induction of 2 membrane transporters. Interestingly, ABC transporter ATP-binding protein (ENTRA1, 109-fold induction) was identified as a protein from *Enterococcus raffinosus* ATCC 49464, while amino acid ABC transporter substrate-binding protein FliY (49-fold induction) belonged to *P. rettgeri* (Figure 3B, Supplementary Table S6). Since the *P. rettgeri* strain used in this study was isolated from a natural habitat, acquisition of the transporter protein from *E. raffinosus* through horizontal transfer cannot be ruled out. The ATP-binding cassette-type vacuolar membrane transporter HMT1 from *Schizosaccharomyces pombe* (SpHMT1) was first shown to be involved in Cd ion import and Cd resistance (Ortiz et al., 1992). Later, it was demonstrated that the ATP-binding cassette transporter HMT1 constitutes a Cd detoxification mechanism that is highly conserved from bacteria to plants to humans (Preveral et al., 2009). Interestingly, ABC transporter ATP-binding protein ENTRA1 (260 amino acids), but not ABC transporter substrate-binding protein FliY, showed homology (ClustalW alignment score 211) to the C-terminal domain (254 amino acids) of SpHMT1 protein (Supplementary Figure S4), suggesting that ENTRA1 could be involved in Cd transport and intracellular accumulation in *P. rettgeri* cells. In *Escherichia coli*, upregulation of the periplasmic L-cystine-binding protein FliY was observed following exposure to 100 μM Cd or 0.88 mM H_2_O_2_ (Helbig et al., 2008; Ohtsu et al., 2010). FliY is an integral component of the L-cysteine/L-cystine shuttle system that imports L-cystine, an oxidized product of L-cysteine, from the periplasm to the cytoplasm to limit lipid peroxidation (Ohtsu et al., 2010). Cells overexpressing cystine/glutamate transporter protein acquire oxidative stress resistance independent of glutathione (GSH) (Banjac et al., 2008). ABC transporter substrate-binding protein FliY (279 amino acids) of *P. rettgeri* showed homology (ClustalW alignment score 398) to the L-cystine-binding protein TcyJ (266 amino acids) of *E. coli* (Supplementary Figure S5). It is, thus, tempting to speculate that the FliY protein provides an additional layer of oxidative stress resistance to Cd-exposed *P. rettgeri* cells. Additionally, an increase in abundance was seen for protein disaggregation chaperone ClpB (5.3-fold) and ATP-dependent protease, and ATPase subunit HslU (1.75-fold) (Figure 3B; Supplementary Table S6). While the organism showed a robust response to alleviate oxidative stress and revive or clear the damaged proteins, GO enrichment analysis on the 46 downregulated DEPs showed enrichment of several metabolic pathways such as the TCA cycle, aerobic respiration, and pyruvate metabolism (Figure 3C, Supplementary Tables S7, S8). Reduced metabolism could be either the direct effect of the diversion of NADH for redox reactions related to Cd bioaccumulation or a strategy adopted by the organism to limit metabolically generated ROS. Zhai et al. (2017) have also reported energy conservation and oxidative stress defense as some of the important mechanisms of Cd tolerance in *Lactobacillus plantarum*. Thus, we conclude that the Cd tolerance of *P. rettgeri* originates from the upregulation of relevant antioxidant enzymes and protein protection proteins.

### Beneficial Effect of *P. rettgeri* on Cd Remediation in Model Rice Ecosystem

**Figure 4:**
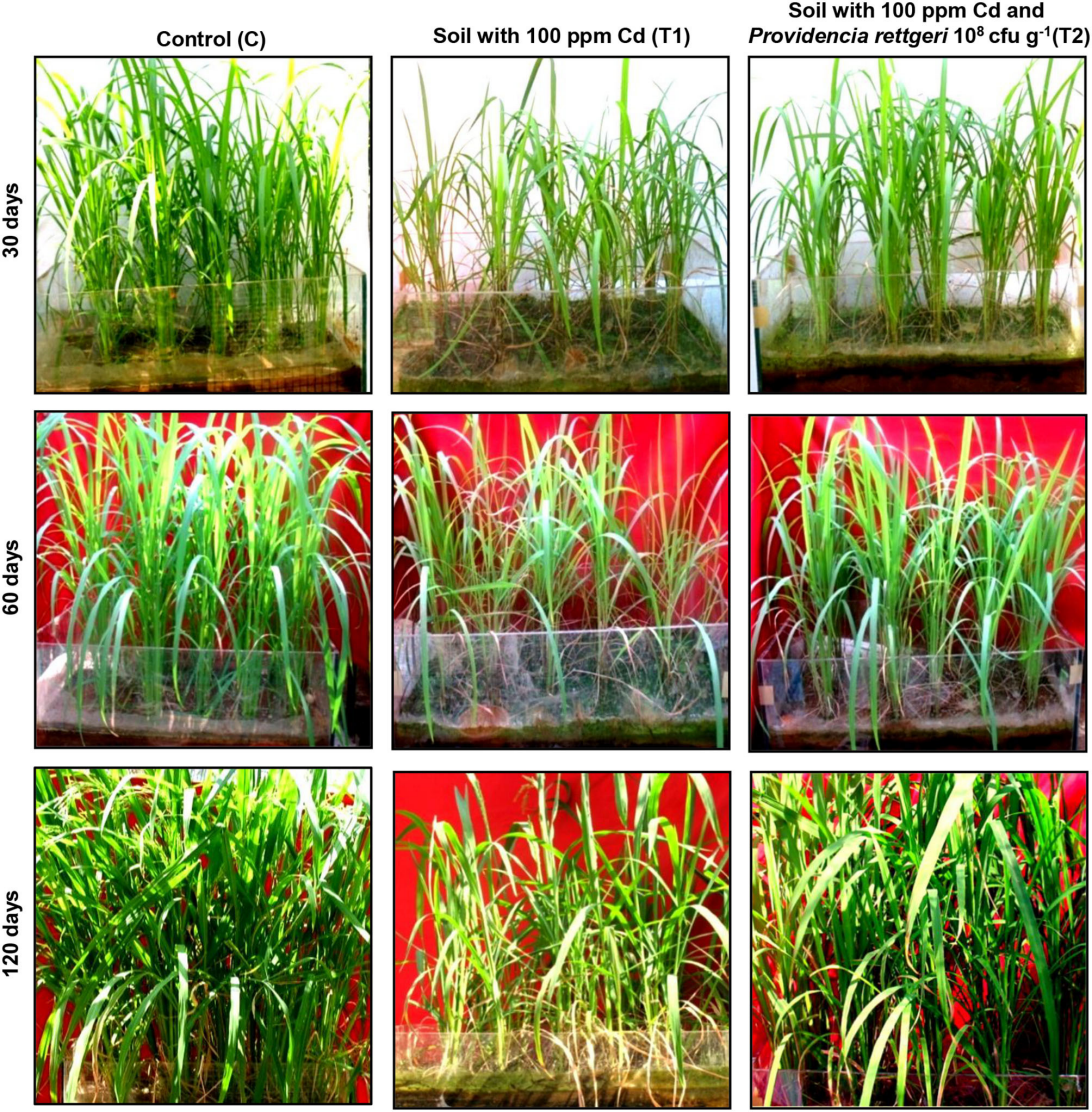
Effect of *P. rettgeri* on Cd toxicity in rice plants. The images show the growth pattern of the rice plants grown either in the absence of Cd (C), in soil spiked with 100 ppm Cd (T1), or in soil spiked with 100 ppm Cd and 10^8^ *P. rettgeri* cells per gram of soil (T2). The images were taken at the different stages of plant development. For larger images, refer to Supplementary Figure S6.

**Figure 5:**
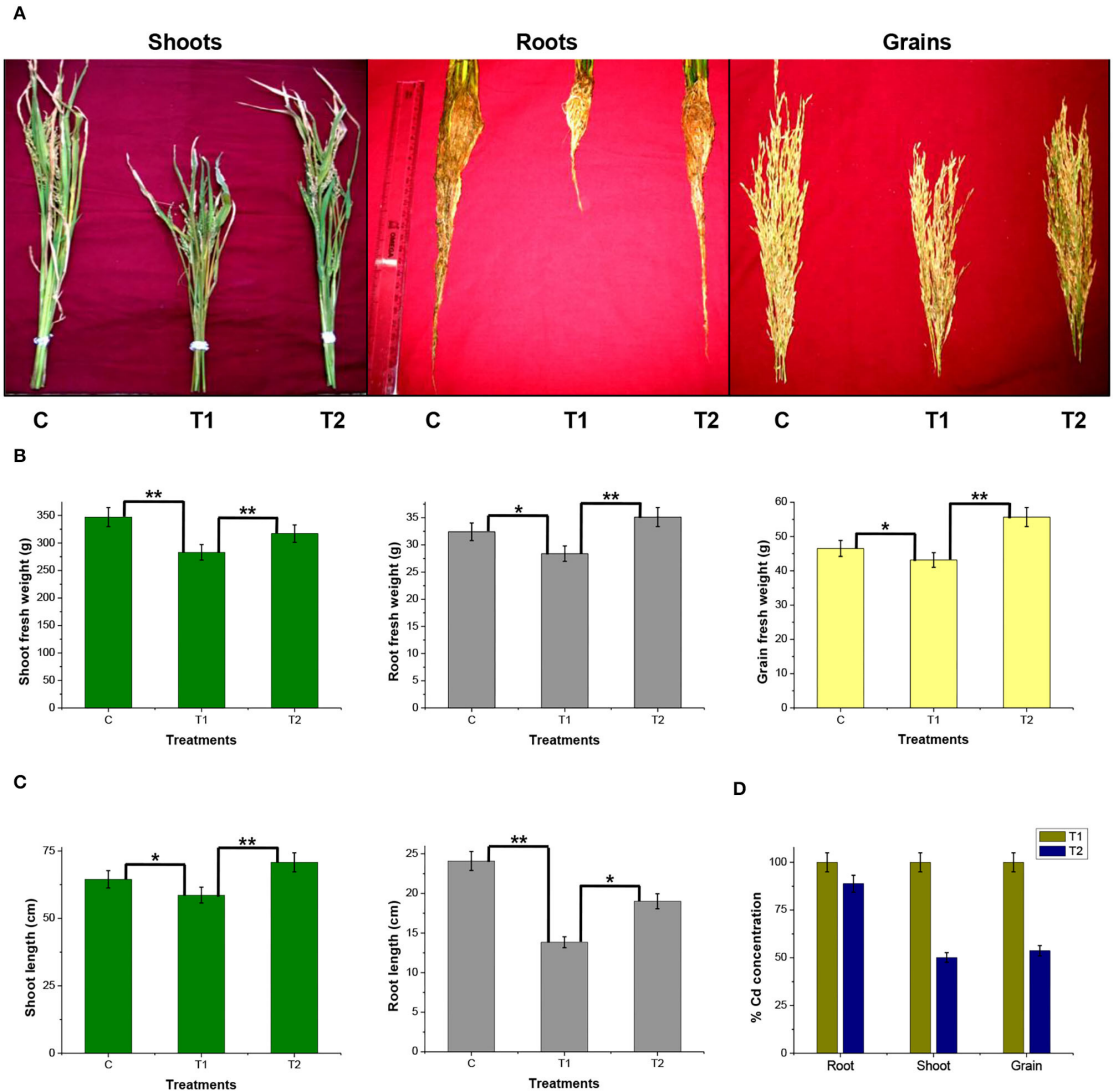
Effect of *P. rettgeri* on different growth parameters of the rice plant. **(A)** The images show shoots, roots, and grains of rice plants harvested at the maturity stage (135 days). **(B)** Fresh weight of or **(C)** length of or **(D)** the Cd concentration in the shoots, roots, or the grains harvested from the rice plants at the maturity stage. Details of C, T1, and T2 are the same as given in Figure 4. The values represent means obtained from three replicates, and the bars represent the standard deviation of means. Comparisons of individual groups were carried out using the *post-hoc* Tukey's test. The *p*-values were obtained using the *post-hoc Q* table. *α ≤ 0.05, i.e., 95% CI; **α ≤ 0.01, i.e., 99% CI based on Tukey's mean grouping. Cd content in control plant tissues was below detectable limits.

A rice ecosystem experiment was performed to evaluate the effect of *P. rettgeri* on different growth parameters of rice plants and the Cd accumulation in different plant parts. The rice plants grown on control soil exhibited normal growth; however, spiking of the soil with 100-ppm Cd resulted in a significant reduction in plant growth throughout the growth phase till 135 days (Figure 4). In contrast, the application of *P. rettgeri* cells to the 100 ppm Cd-spiked soil improved the growth of rice plants on par with control plants, demonstrating that the presence of *P. rettgeri* cells had a beneficial effect on reducing Cd toxicity (Figure 4). Furthermore, we found that the fresh weights of roots, shoots, and grains of rice plants grown in the presence of 100 ppm Cd (T1) were significantly reduced as compared to control (C) plants (Figures 5A,B). The application of *P. rettgeri* cells to 100 ppm Cd containing soil (T2) caused a significant increase in the fresh weights of roots, shoots, and grains as compared to plants grown in the presence of 100 ppm Cd (T1). The plants exposed to 100 ppm Cd (T1) showed a significant reduction in the elongation of shoots and roots as compared to control plants (C) (Figure 5C). However, the application of *P. rettgeri* cells to 100 ppm Cd-containing soil (T2) nullified the toxic effects of Cd on the elongation of shoots and roots (Figure 5C). Cd contents in the control plant parts were below detectable limits. In the plants exposed to 100 ppm Cd (T1), roots accumulated the highest amount of Cd (575 ppm), followed by shoots (90 ppm) and grains (36 ppm). When 100 ppm Cd soil was treated with *P. rettgeri*, Cd content in the roots and the shoots were reduced by 11.2 and 50%, respectively, while the grains showed a 46.1% reduction (Figure 5D). Our results demonstrate that the presence of *P. rettgeri* cells has synergistic effects on minimizing Cd toxicity in the rice ecosystem.

*In-situ* bioremediation of soil has gained importance in recent years. To access the feasibility of the application of any metal-tolerant bacteria at the field level, it is necessary to simulate the field conditions and study its potential for *in-situ* bioremediation. This is the first report of its kind wherein a rice field condition is simulated and developed in the form of a rice ecosystem and the potential of *P. rettgeri* bacteria for Cd *in-situ* bioremediation is studied in crops grown till full maturity (135 days). The overall outcome of the study has indicated that the Cd bioaccumulation property of *P. rettgeri* cells has favorably decreased the Cd contents (~50%) in the edible parts of the rice plant due to reduced phytoavailability of Cd in soils. This is in agreement with *in-situ* bioremediation reported using other microbes (Chi et al., 2020; Haider et al., 2021). Various strains such as *Enterobacter aerogenes* MCC 3092, *Pseudomonas aeruginosa, Bacillus megaterium* H3, and *Neorhizobium huautlense* T1-17 were found to be potentially useful microbes in reducing phytoavailable Cd from the soil in the range of 40–60% Cd in different rice plant parts (Lin et al., 2016; Pramanik et al., 2018; Zhou et al., 2021).

## Conclusion

**Figure 6:**
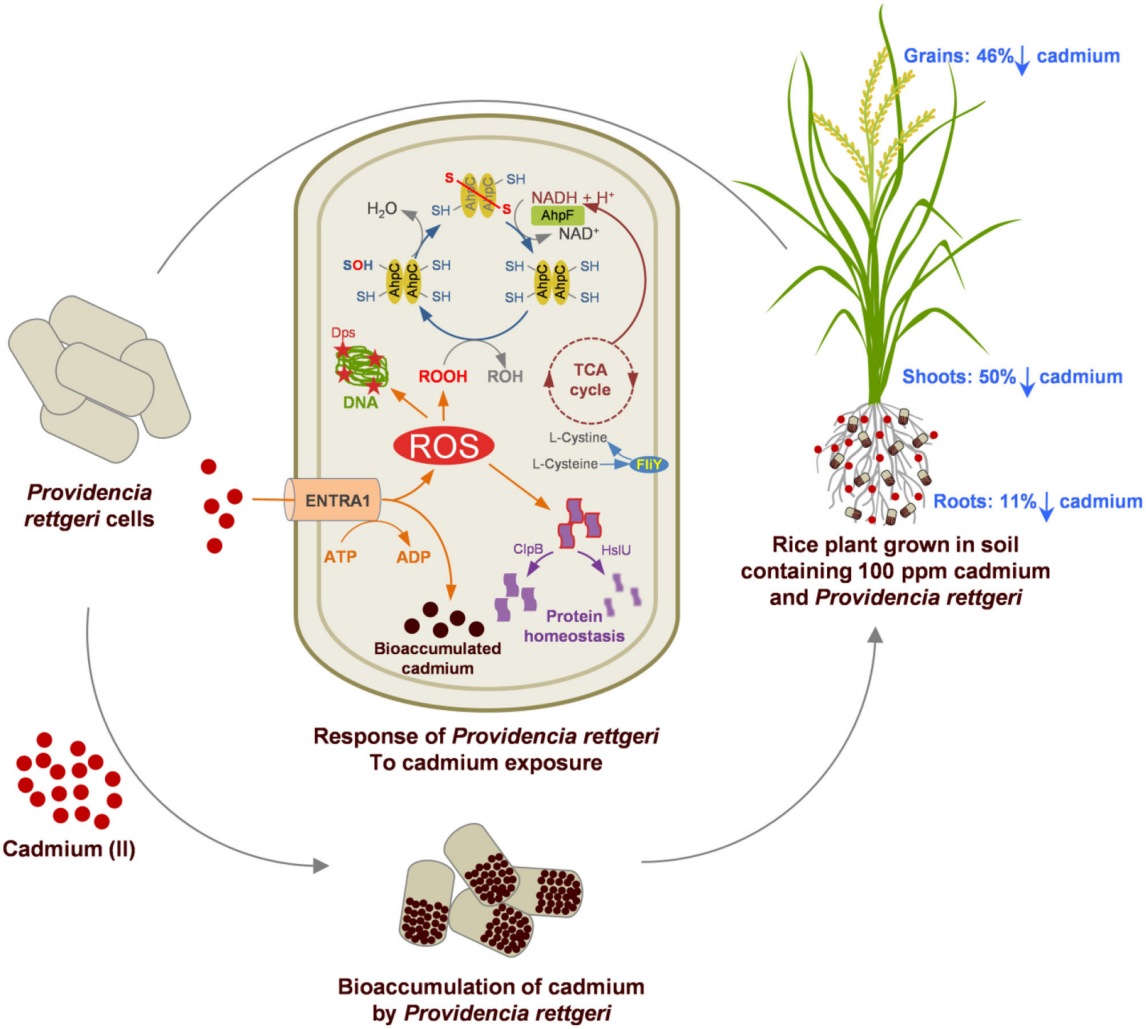
A proteome and rice ecosystem-based model for the Cd tolerance and *in-situ* bioremediation potential of *P. rettgeri* KDM3 strain. Red dots represent Cd present in the soil, and brown dots indicate intracellularly accumulated Cd in the *P. rettgeri* cells. Percentage reduction in cadmium concentration in roots, shoots, and grains is shown by down arrows. The model at the center depicts the Cd responsive proteins involved in Cd tolerance and their possible role in Cd accumulation and cellular homeostasis. For details of Cd responsive proteins, refer to Supplementary Table S2.

The Cd tolerance of *P. rettgeri* and its potential for *in-situ* bioremediation are summarized in Figure 6. Cd-tolerant *P. rettgeri* bioaccumulated Cd through molecular mechanisms for Cd transport (ENTRA1), oxidative stress alleviation (AhpCF, Dps, FliY, and SodA), and proteome protection (ClpB and HslU). This facultative aerobe was further demonstrated to be most suitable to the unique environment offered by the submerged conditions of the paddy. The presence of *P. rettgeri* significantly decreased Cd concentrations in root, shoot, and grain due to reduced Cd mobility in the soil. Bioaugmentation of Cd-contaminated soil with *P. rettgeri* offers a solution for masking Cd toxicity in the rice plants by improving overall growth till maturity and yielding low-Cd rice.

## Data Availability Statement

The datasets presented in this study can be found in online repositories. The names of the repository/repositories and accession number(s) can be found in the article/Supplementary Material.

## Author Contributions

DS: designed the experiments, carried out the experiments, analyzed the data, and written and finalized the manuscript. SK: conceptualized the project. BB: carried out proteomics experiments, analyzed the data, and written and finalized the manuscript. AG: carried out TEM experiments and analyzed data. MP: analyzed the proteomics data. All authors contributed to the article and approved the submitted version.

## Conflict of Interest

The authors declare that the research was conducted in the absence of any commercial or financial relationships that could be construed as a potential conflict of interest.

## Publisher's Note

All claims expressed in this article are solely those of the authors and do not necessarily represent those of their affiliated organizations, or those of the publisher, the editors and the reviewers. Any product that may be evaluated in this article, or claim that may be made by its manufacturer, is not guaranteed or endorsed by the publisher.
