# Supplementary material for: Proteomic Perspective of Cadmium Tolerance in Providencia rettgeri Strain KDM3 and Its In-situ Bioremediation Potential in Rice Ecosystem
Source: Front Microbiol. 2022 Apr 26;13:852697. doi: 10.3389/fmicb.2022.852697 (PMC9086847; doi:10.3389/fmicb.2022.852697)
Supplement: Supplementary file 3 [file Presentation1.PPTX]

## Slide 1
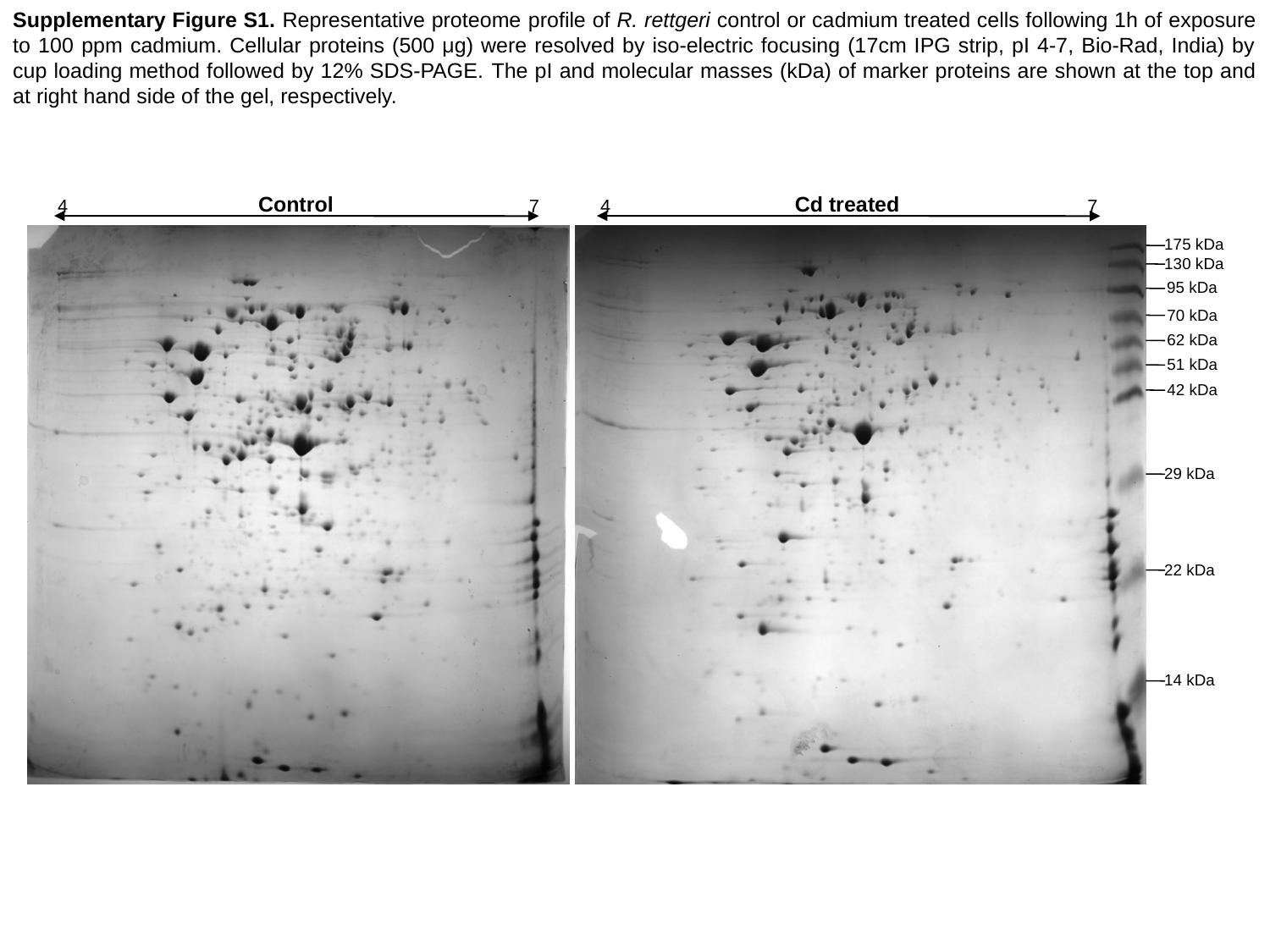

Supplementary Figure S1. Representative proteome profile of R. rettgeri control or cadmium treated cells following 1h of exposure to 100 ppm cadmium. Cellular proteins (500 μg) were resolved by iso-electric focusing (17cm IPG strip, pI 4-7, Bio-Rad, India) by cup loading method followed by 12% SDS-PAGE. The pI and molecular masses (kDa) of marker proteins are shown at the top and at right hand side of the gel, respectively.
Control
Cd treated
4
7
4
7
175 kDa
130 kDa
95 kDa
70 kDa
62 kDa
51 kDa
42 kDa
29 kDa
22 kDa
14 kDa

## Slide 2
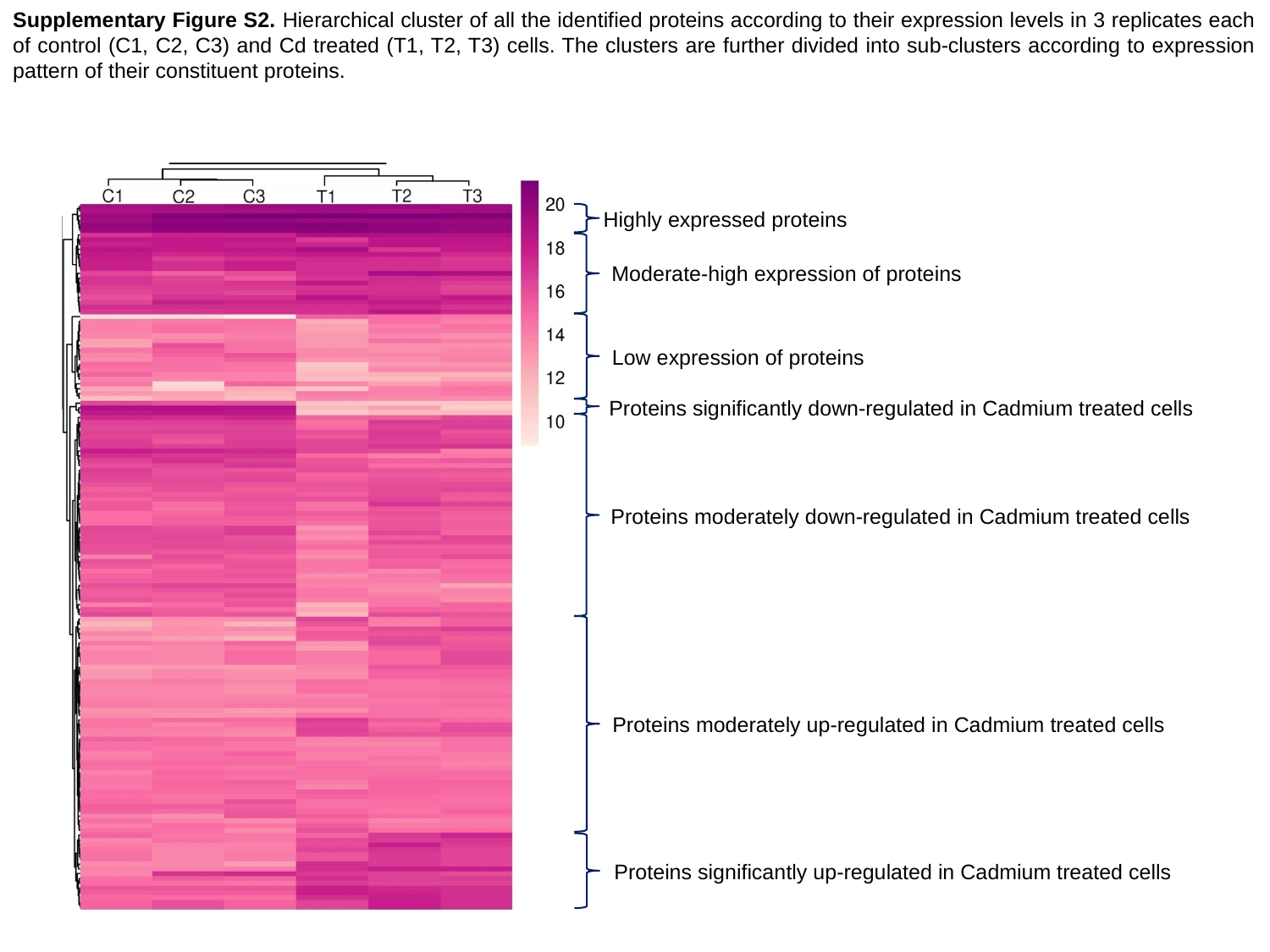

Supplementary Figure S2. Hierarchical cluster of all the identified proteins according to their expression levels in 3 replicates each of control (C1, C2, C3) and Cd treated (T1, T2, T3) cells. The clusters are further divided into sub-clusters according to expression pattern of their constituent proteins.
Highly expressed proteins
Moderate-high expression of proteins
Low expression of proteins
Proteins significantly down-regulated in Cadmium treated cells
Proteins moderately down-regulated in Cadmium treated cells
Proteins moderately up-regulated in Cadmium treated cells
Proteins significantly up-regulated in Cadmium treated cells

## Slide 3
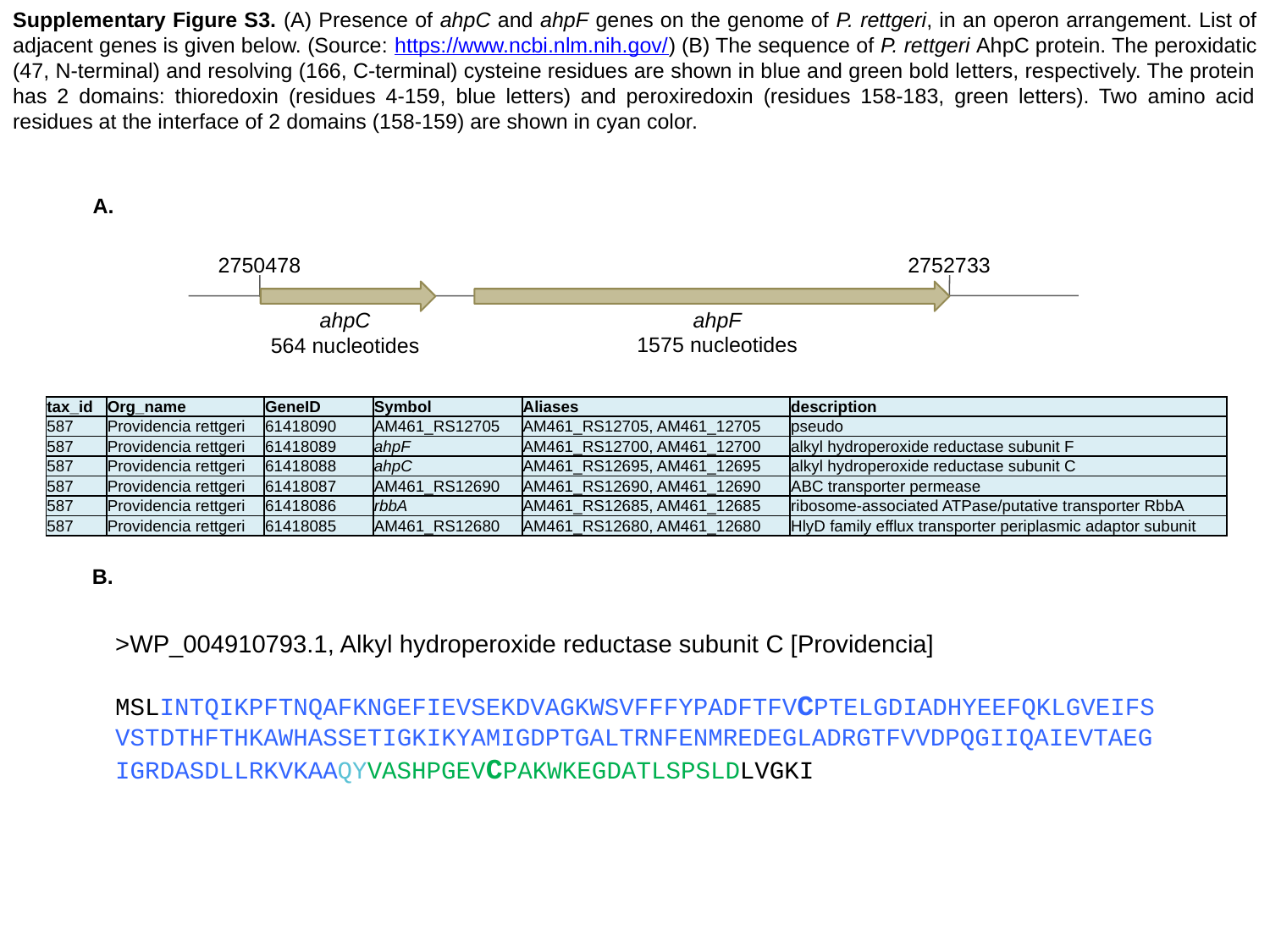

Supplementary Figure S3. (A) Presence of ahpC and ahpF genes on the genome of P. rettgeri, in an operon arrangement. List of adjacent genes is given below. (Source: https://www.ncbi.nlm.nih.gov/) (B) The sequence of P. rettgeri AhpC protein. The peroxidatic (47, N-terminal) and resolving (166, C-terminal) cysteine residues are shown in blue and green bold letters, respectively. The protein has 2 domains: thioredoxin (residues 4-159, blue letters) and peroxiredoxin (residues 158-183, green letters). Two amino acid residues at the interface of 2 domains (158-159) are shown in cyan color.
A.
2750478
2752733
ahpF
1575 nucleotides
ahpC
564 nucleotides
| tax\_id | Org\_name | GeneID | Symbol | Aliases | description |
| --- | --- | --- | --- | --- | --- |
| 587 | Providencia rettgeri | 61418090 | AM461\_RS12705 | AM461\_RS12705, AM461\_12705 | pseudo |
| 587 | Providencia rettgeri | 61418089 | ahpF | AM461\_RS12700, AM461\_12700 | alkyl hydroperoxide reductase subunit F |
| 587 | Providencia rettgeri | 61418088 | ahpC | AM461\_RS12695, AM461\_12695 | alkyl hydroperoxide reductase subunit C |
| 587 | Providencia rettgeri | 61418087 | AM461\_RS12690 | AM461\_RS12690, AM461\_12690 | ABC transporter permease |
| 587 | Providencia rettgeri | 61418086 | rbbA | AM461\_RS12685, AM461\_12685 | ribosome-associated ATPase/putative transporter RbbA |
| 587 | Providencia rettgeri | 61418085 | AM461\_RS12680 | AM461\_RS12680, AM461\_12680 | HlyD family efflux transporter periplasmic adaptor subunit |
B.
>WP_004910793.1, Alkyl hydroperoxide reductase subunit C [Providencia]
MSLINTQIKPFTNQAFKNGEFIEVSEKDVAGKWSVFFFYPADFTFVCPTELGDIADHYEEFQKLGVEIFS
VSTDTHFTHKAWHASSETIGKIKYAMIGDPTGALTRNFENMREDEGLADRGTFVVDPQGIIQAIEVTAEG
IGRDASDLLRKVKAAQYVASHPGEVCPAKWKEGDATLSPSLDLVGKI

## Slide 4
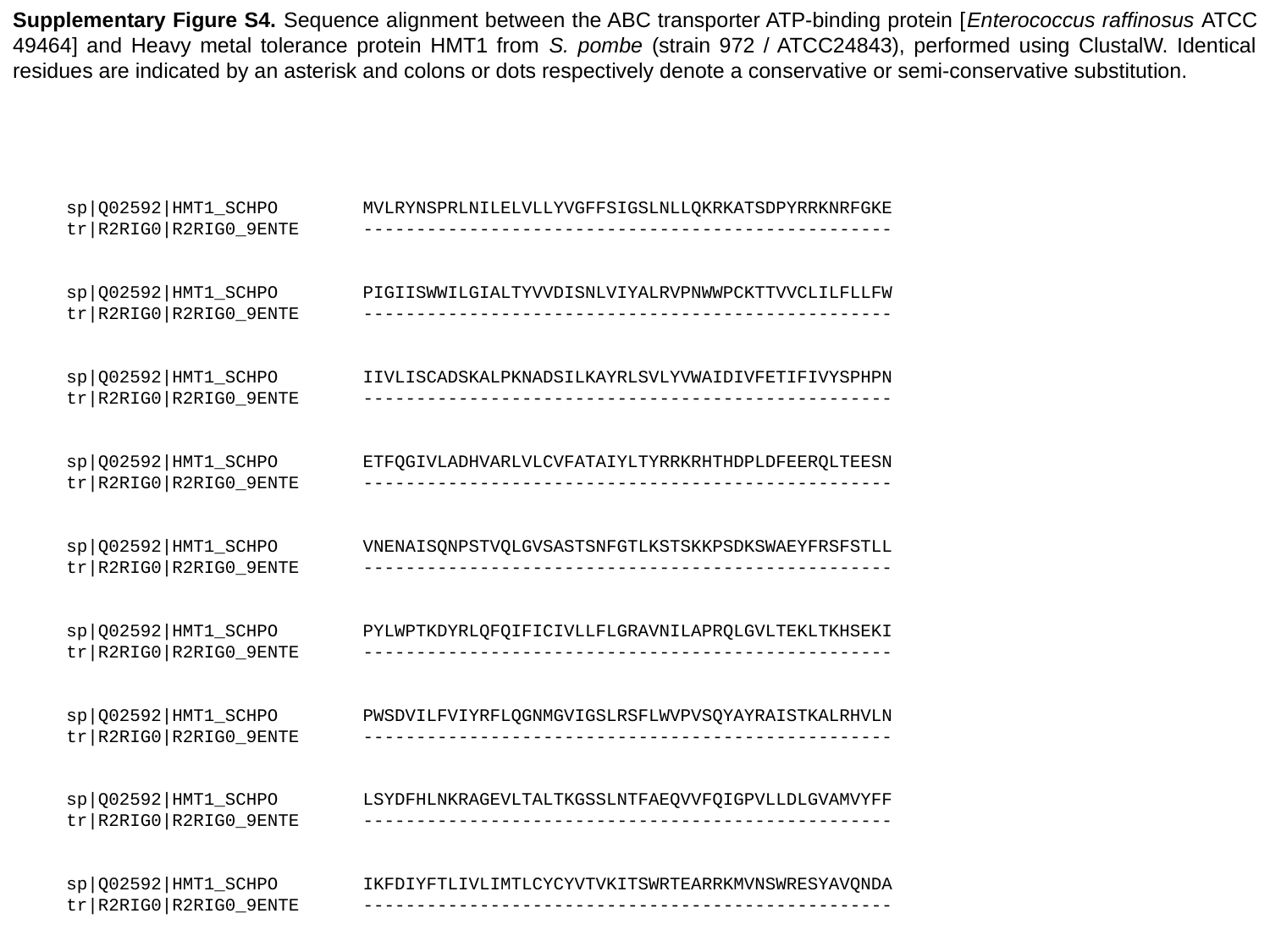

Supplementary Figure S4. Sequence alignment between the ABC transporter ATP-binding protein [Enterococcus raffinosus ATCC 49464] and Heavy metal tolerance protein HMT1 from S. pombe (strain 972 / ATCC24843), performed using ClustalW. Identical residues are indicated by an asterisk and colons or dots respectively denote a conservative or semi-conservative substitution.
sp|Q02592|HMT1_SCHPO MVLRYNSPRLNILELVLLYVGFFSIGSLNLLQKRKATSDPYRRKNRFGKE
tr|R2RIG0|R2RIG0_9ENTE --------------------------------------------------
sp|Q02592|HMT1_SCHPO PIGIISWWILGIALTYVVDISNLVIYALRVPNWWPCKTTVVCLILFLLFW
tr|R2RIG0|R2RIG0_9ENTE --------------------------------------------------
sp|Q02592|HMT1_SCHPO IIVLISCADSKALPKNADSILKAYRLSVLYVWAIDIVFETIFIVYSPHPN
tr|R2RIG0|R2RIG0_9ENTE --------------------------------------------------
sp|Q02592|HMT1_SCHPO ETFQGIVLADHVARLVLCVFATAIYLTYRRKRHTHDPLDFEERQLTEESN
tr|R2RIG0|R2RIG0_9ENTE --------------------------------------------------
sp|Q02592|HMT1_SCHPO VNENAISQNPSTVQLGVSASTSNFGTLKSTSKKPSDKSWAEYFRSFSTLL
tr|R2RIG0|R2RIG0_9ENTE --------------------------------------------------
sp|Q02592|HMT1_SCHPO PYLWPTKDYRLQFQIFICIVLLFLGRAVNILAPRQLGVLTEKLTKHSEKI
tr|R2RIG0|R2RIG0_9ENTE --------------------------------------------------
sp|Q02592|HMT1_SCHPO PWSDVILFVIYRFLQGNMGVIGSLRSFLWVPVSQYAYRAISTKALRHVLN
tr|R2RIG0|R2RIG0_9ENTE --------------------------------------------------
sp|Q02592|HMT1_SCHPO LSYDFHLNKRAGEVLTALTKGSSLNTFAEQVVFQIGPVLLDLGVAMVYFF
tr|R2RIG0|R2RIG0_9ENTE --------------------------------------------------
sp|Q02592|HMT1_SCHPO IKFDIYFTLIVLIMTLCYCYVTVKITSWRTEARRKMVNSWRESYAVQNDA
tr|R2RIG0|R2RIG0_9ENTE --------------------------------------------------

## Slide 5
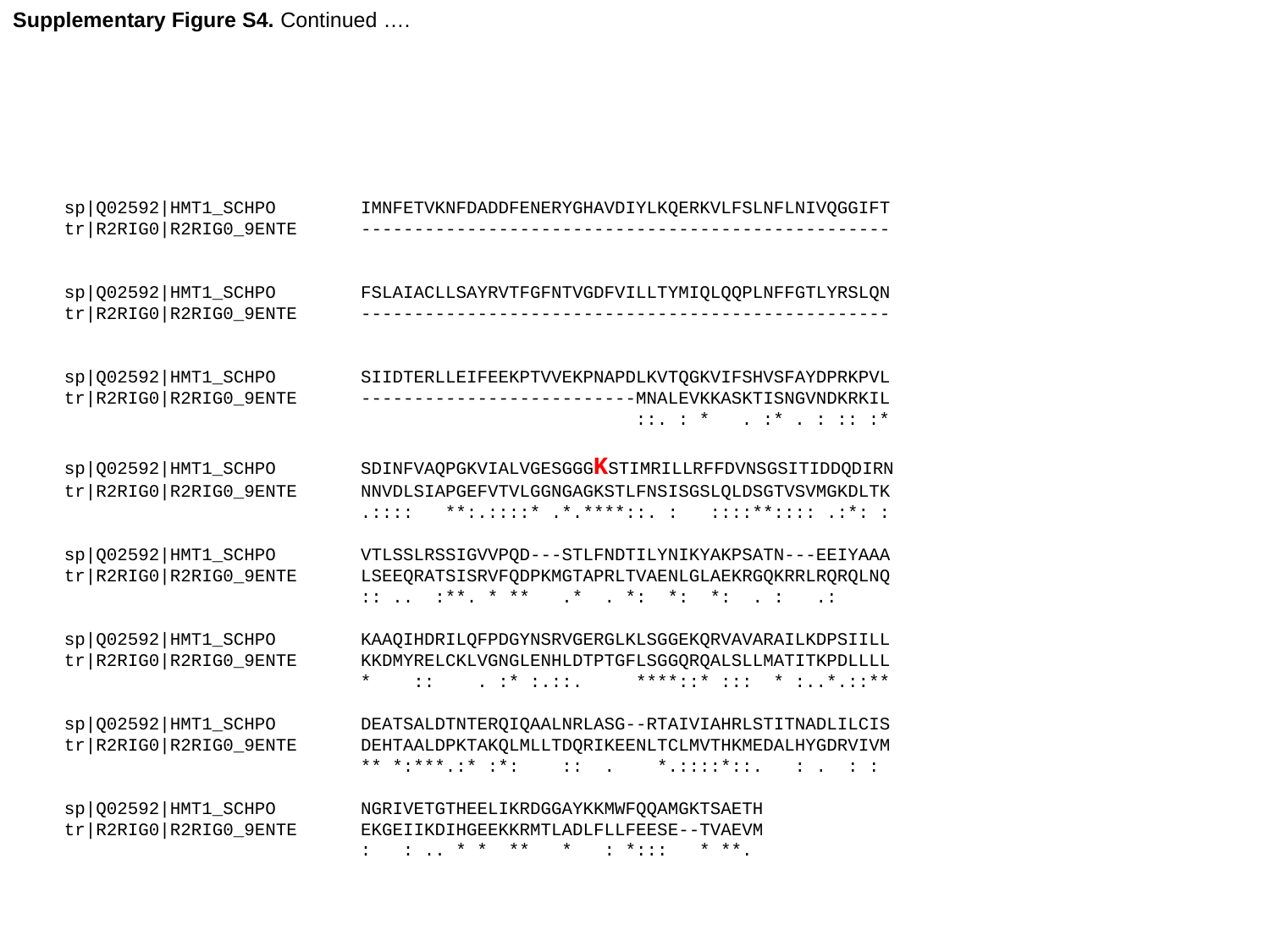

Supplementary Figure S4. Continued ….
sp|Q02592|HMT1_SCHPO IMNFETVKNFDADDFENERYGHAVDIYLKQERKVLFSLNFLNIVQGGIFT
tr|R2RIG0|R2RIG0_9ENTE --------------------------------------------------
sp|Q02592|HMT1_SCHPO FSLAIACLLSAYRVTFGFNTVGDFVILLTYMIQLQQPLNFFGTLYRSLQN
tr|R2RIG0|R2RIG0_9ENTE --------------------------------------------------
sp|Q02592|HMT1_SCHPO SIIDTERLLEIFEEKPTVVEKPNAPDLKVTQGKVIFSHVSFAYDPRKPVL
tr|R2RIG0|R2RIG0_9ENTE --------------------------MNALEVKKASKTISNGVNDKRKIL
 ::. : * . :* . : :: :*
sp|Q02592|HMT1_SCHPO SDINFVAQPGKVIALVGESGGGKSTIMRILLRFFDVNSGSITIDDQDIRN
tr|R2RIG0|R2RIG0_9ENTE NNVDLSIAPGEFVTVLGGNGAGKSTLFNSISGSLQLDSGTVSVMGKDLTK
 .:::: **:.::::* .*.****::. : ::::**:::: .:*: :
sp|Q02592|HMT1_SCHPO VTLSSLRSSIGVVPQD---STLFNDTILYNIKYAKPSATN---EEIYAAA
tr|R2RIG0|R2RIG0_9ENTE LSEEQRATSISRVFQDPKMGTAPRLTVAENLGLAEKRGQKRRLRQRQLNQ
 :: .. :**. * ** .* . *: *: *: . : .:
sp|Q02592|HMT1_SCHPO KAAQIHDRILQFPDGYNSRVGERGLKLSGGEKQRVAVARAILKDPSIILL
tr|R2RIG0|R2RIG0_9ENTE KKDMYRELCKLVGNGLENHLDTPTGFLSGGQRQALSLLMATITKPDLLLL
 * :: . :* :.::. ****::* ::: * :..*.::**
sp|Q02592|HMT1_SCHPO DEATSALDTNTERQIQAALNRLASG--RTAIVIAHRLSTITNADLILCIS
tr|R2RIG0|R2RIG0_9ENTE DEHTAALDPKTAKQLMLLTDQRIKEENLTCLMVTHKMEDALHYGDRVIVM
 ** *:***.:* :*: :: . *.::::*::. : . : :
sp|Q02592|HMT1_SCHPO NGRIVETGTHEELIKRDGGAYKKMWFQQAMGKTSAETH
tr|R2RIG0|R2RIG0_9ENTE EKGEIIKDIHGEEKKRMTLADLFLLFEESE--TVAEVM
 : : .. * * ** * : *::: * **.

## Slide 6
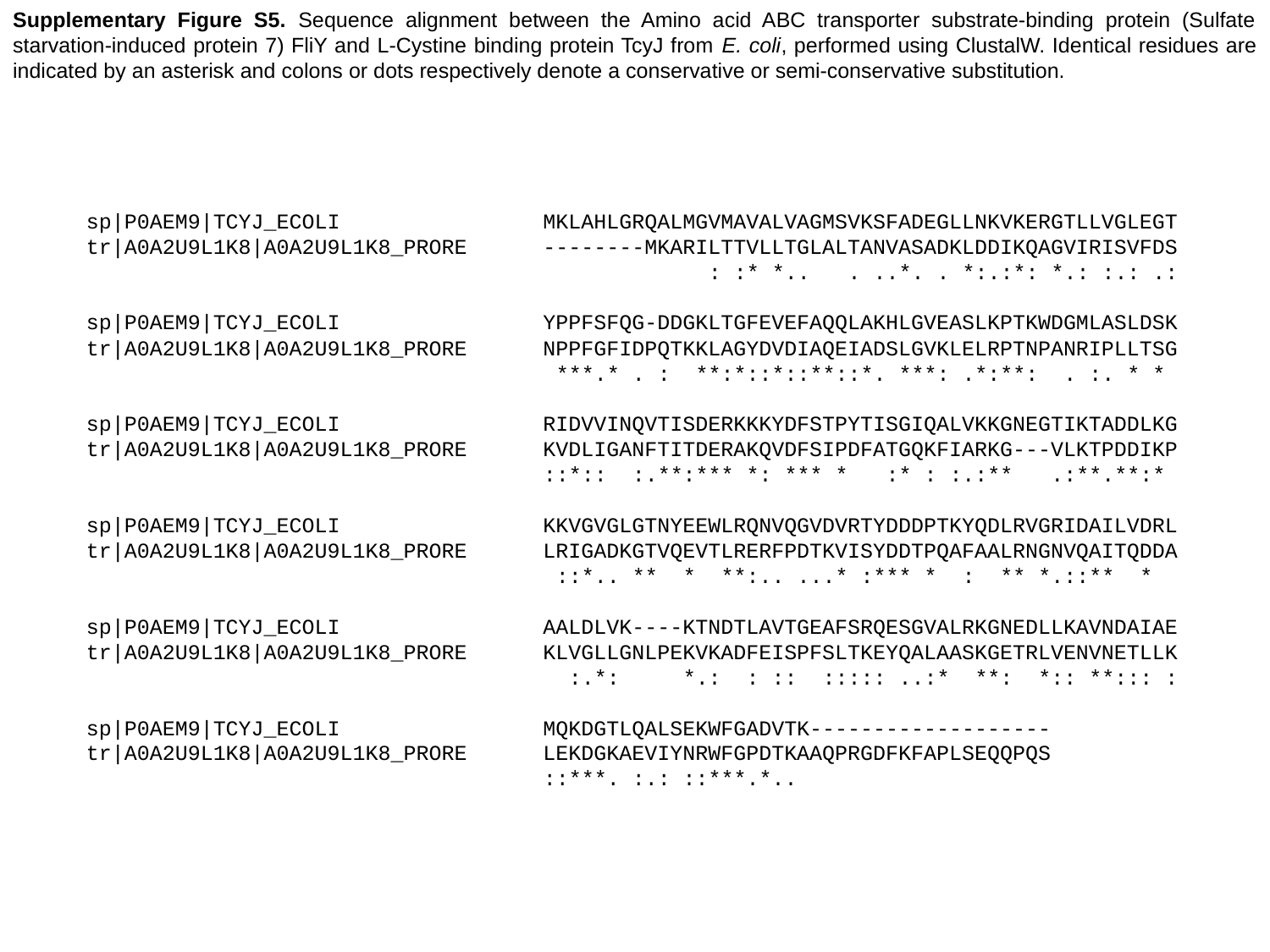

Supplementary Figure S5. Sequence alignment between the Amino acid ABC transporter substrate-binding protein (Sulfate starvation-induced protein 7) FliY and L-Cystine binding protein TcyJ from E. coli, performed using ClustalW. Identical residues are indicated by an asterisk and colons or dots respectively denote a conservative or semi-conservative substitution.
sp|P0AEM9|TCYJ_ECOLI MKLAHLGRQALMGVMAVALVAGMSVKSFADEGLLNKVKERGTLLVGLEGT
tr|A0A2U9L1K8|A0A2U9L1K8_PRORE --------MKARILTTVLLTGLALTANVASADKLDDIKQAGVIRISVFDS
 : :* *.. . ..*. . *:.:*: *.: :.: .:
sp|P0AEM9|TCYJ_ECOLI YPPFSFQG-DDGKLTGFEVEFAQQLAKHLGVEASLKPTKWDGMLASLDSK
tr|A0A2U9L1K8|A0A2U9L1K8_PRORE NPPFGFIDPQTKKLAGYDVDIAQEIADSLGVKLELRPTNPANRIPLLTSG
 ***.* . : **:*::*::**::*. ***: .*:**: . :. * *
sp|P0AEM9|TCYJ_ECOLI RIDVVINQVTISDERKKKYDFSTPYTISGIQALVKKGNEGTIKTADDLKG
tr|A0A2U9L1K8|A0A2U9L1K8_PRORE KVDLIGANFTITDERAKQVDFSIPDFATGQKFIARKG---VLKTPDDIKP
 ::*:: :.**:*** *: *** * :* : :.:** .:**.**:*
sp|P0AEM9|TCYJ_ECOLI KKVGVGLGTNYEEWLRQNVQGVDVRTYDDDPTKYQDLRVGRIDAILVDRL
tr|A0A2U9L1K8|A0A2U9L1K8_PRORE LRIGADKGTVQEVTLRERFPDTKVISYDDTPQAFAALRNGNVQAITQDDA
 ::*.. ** * **:.. ...* :*** * : ** *.::** *
sp|P0AEM9|TCYJ_ECOLI AALDLVK----KTNDTLAVTGEAFSRQESGVALRKGNEDLLKAVNDAIAE
tr|A0A2U9L1K8|A0A2U9L1K8_PRORE KLVGLLGNLPEKVKADFEISPFSLTKEYQALAASKGETRLVENVNETLLK
 :.*: *.: : :: ::::: ..:* **: *:: **::: :
sp|P0AEM9|TCYJ_ECOLI MQKDGTLQALSEKWFGADVTK-------------------
tr|A0A2U9L1K8|A0A2U9L1K8_PRORE LEKDGKAEVIYNRWFGPDTKAAQPRGDFKFAPLSEQQPQS
 ::***. :.: ::***.*..

## Slide 7
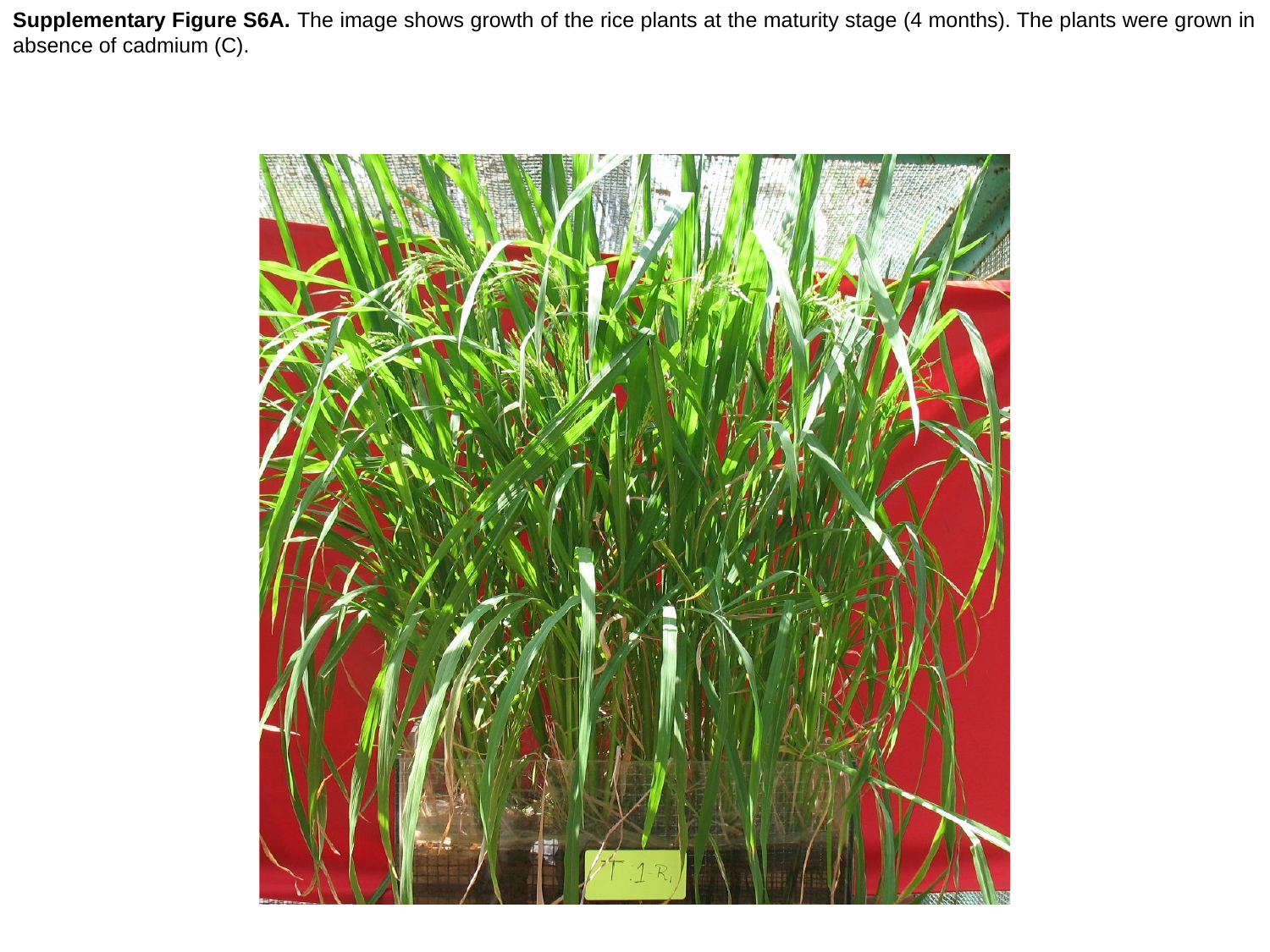

Supplementary Figure S6A. The image shows growth of the rice plants at the maturity stage (4 months). The plants were grown in absence of cadmium (C).

## Slide 8
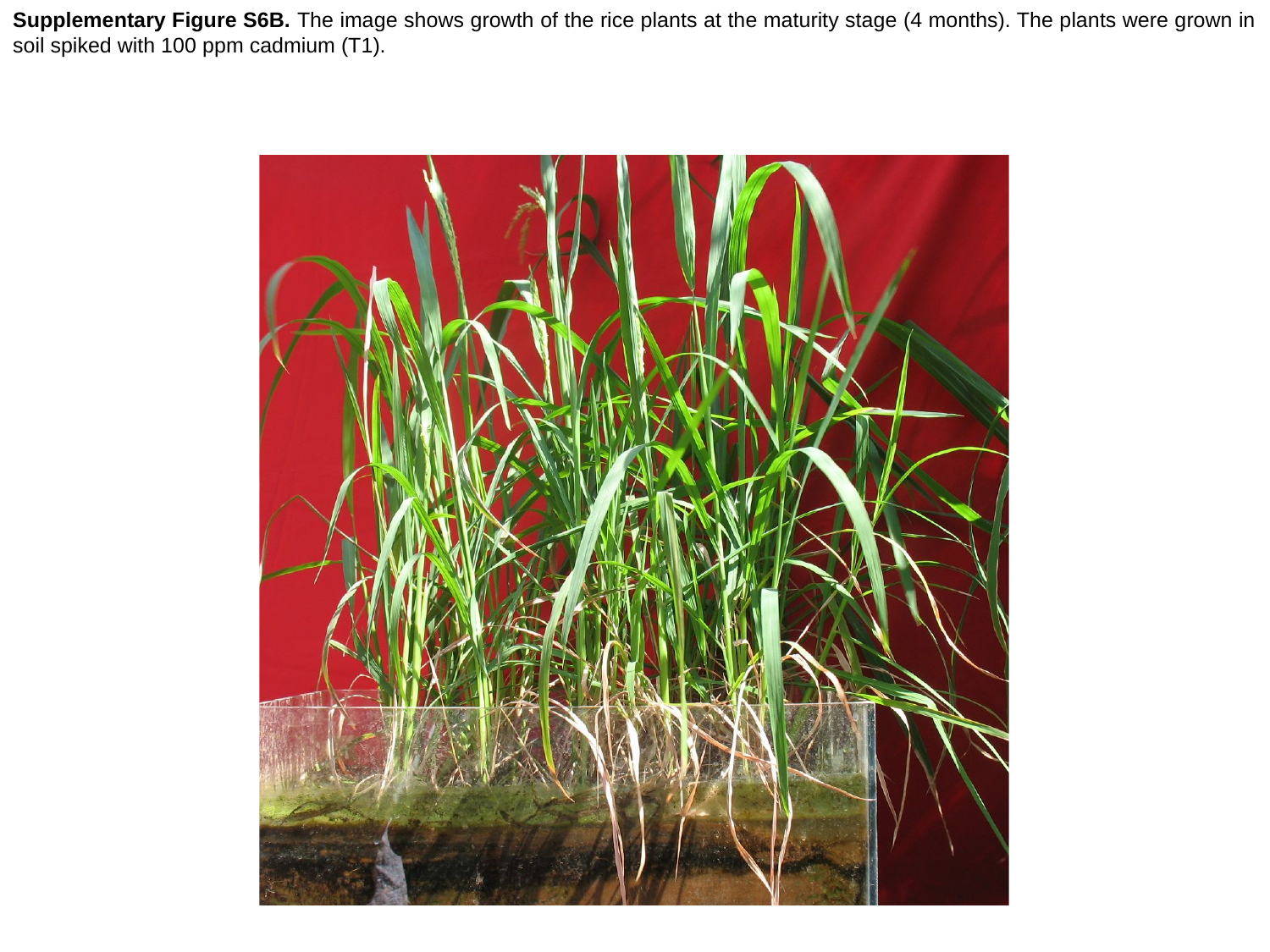

Supplementary Figure S6B. The image shows growth of the rice plants at the maturity stage (4 months). The plants were grown in soil spiked with 100 ppm cadmium (T1).

## Slide 9
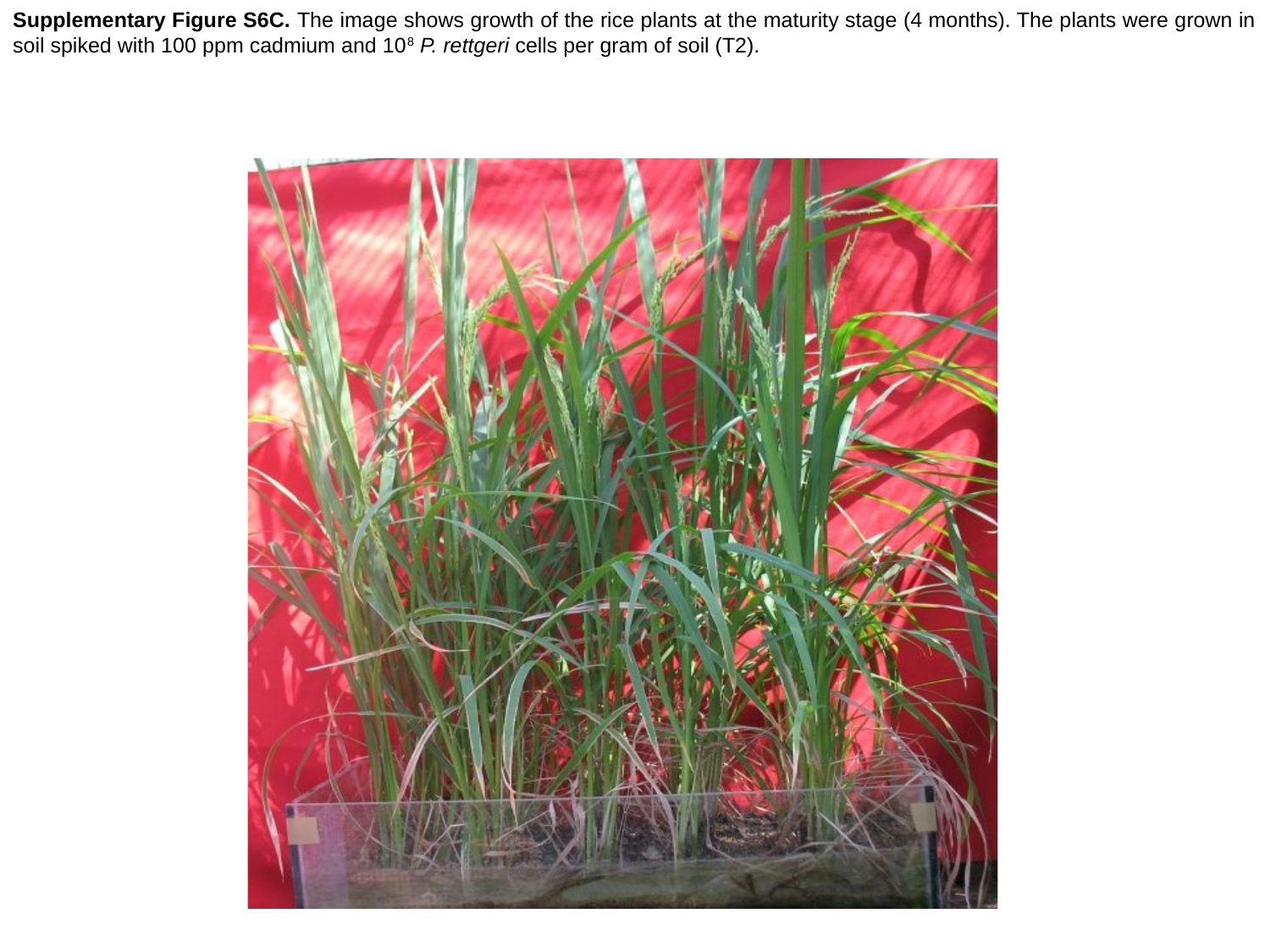

Supplementary Figure S6C. The image shows growth of the rice plants at the maturity stage (4 months). The plants were grown in soil spiked with 100 ppm cadmium and 108 P. rettgeri cells per gram of soil (T2).
